# Mechanisms of gefitinib-induced interstitial pneumonitis: why and how the TKI perturbs innate immune systems?

**DOI:** 10.18632/oncotarget.27958

**Published:** 2021-06-22

**Authors:** Tomohiro Kagi, Takuya Noguchi, Atsushi Matsuzawa

**Keywords:** gefitinib, interstitial pneumonitis, tyrosine kinase inhibitor, inflammation, NLRP3 inflammasome

Gefitinib is a representative tyrosine kinase inhibitor (TKI) that blocks the tyrosine kinase activity of epidermal growth factor receptor (EGFR), and thereby exerts anticancer activity against various types of solid tumors, including inoperable non-small cell lung cancer with EGFR mutation [1]. Although gefitinib is a molecular target drug, several studies have revealed that gefitinib targets unexpected proteins besides EGFR, which may be associated with the serious side effects of gefitinib, such as interstitial pneumonitis [1–3]. Interstitial pneumonia refers to potent inflammation of lung tissue caused by over-activation of the immune system triggered by drugs or viral infections, resulting in dyspnea and respiratory failure. In particular, infiltration of immune cells into the interstitium and concomitant increase in the amount of pro-inflammatory cytokines are known to play an important role in pathogenesis of interstitial pneumonia [4]. Therefore, to elucidate mechanisms of gefitinib-induced inflammation is an effective approach to overcome interstitial pneumonia initiated by gefitinib.

We have recently demonstrated that gefitinib markedly increases the production of IL-1β in macrophages by stimulating the NLRP3 inflammasome, and MCC950, the specific inhibitor of the NLRP3 (NACHT, LRR and PYD-containing protein 3) inflammasome, clearly suppresses the lung inflammation triggered by gefitinib *in vivo*, suggesting that the NLRP3 inflammasome plays a critical role in the initiation of interstitial pneumonitis [5]. To date, several causal factors of the NLRP3 inflammasome activation have been proposed, such as potassium efflux, calcium overload, and mitochondrial reactive oxidative species (ROS) [6]. Interestingly, our study has shown that gefitinib causes mitochondrial damage followed by production of mitochondrial ROS, which is responsible for the activation on the NLRP3 inflammasome. However, the mechanisms by which gefitinib initiates mitochondrial damage have remained challenging. In this regard, there are two possible mechanisms by which gefitinib produces mitochondrial ROS. One is that gefitinib structurally damages mitochondria, and then accumulation of damaged mitochondria triggers the activation of the NLRP3 inflammasome [7]. To investigate this possibility, other TKIs that have different structures from gefitinib should be comprehensively tested whether they can activate the NLRP3 inflammasome. The other is that nonspecific inhibition of tyrosine kinases by gefitinib disrupts mitochondrial homeostasis. Indeed, it is known that several tyrosine kinases participate in maintenance of mitochondrial homeostasis by regulating the mitochondrial electron transfer system [8, 9]. Therefore, the off-target effects of gefitinib on mitochondrial tyrosine kinases may disrupt mitochondrial homeostasis. In this case, to identify the mitochondrial tyrosine kinases that are sensitive to gefitinib may lead to understanding causal mechanisms of gefitinib-induced inflammation.

We have also demonstrated that gefitinib promotes high-mobility group box 1 (HMGB1) release [5]. The release of HMGB1 promotes a positive feedback loop of the NLRP3 inflammasome activation, leading to excessive inflammation. HMGB1 therefore has emerged as a key mediator of inflammation. Mechanistically, it is interesting that gefitinib can actively release HMGB1 without the NLRP3 inflammasome. Gefitinib-induced mitochondrial ROS elicit DNA damage, which activates poly (ADP-ribose) polymerase-1 (PARP-1). Then, activated PARP-1 seems to promote the HMGB1 release via poly-ADP-ribosylation of HMGB1 [5].

**Figure 1:**
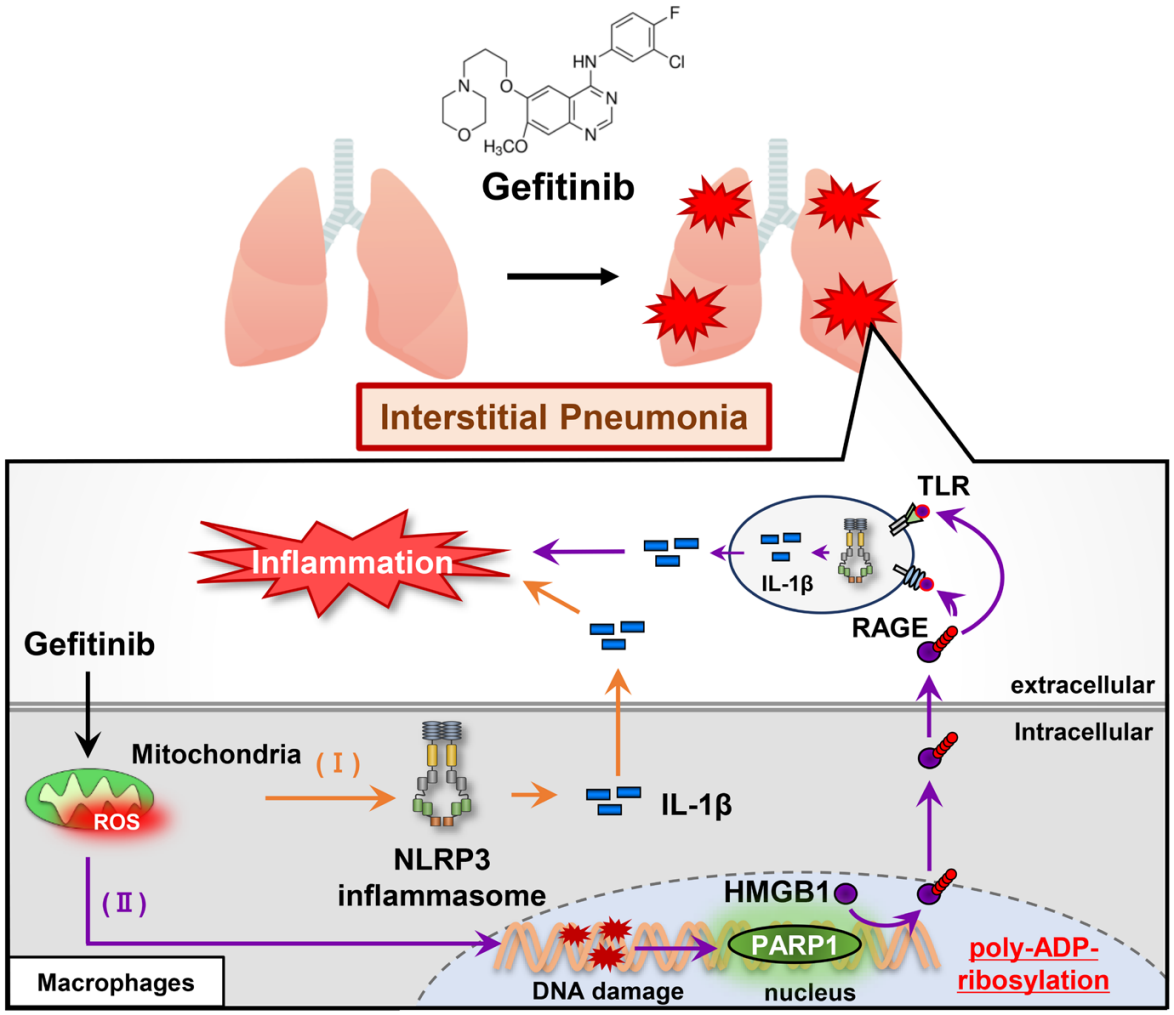
Gefitinib initiates sterile inflammation via two distinct mechanisms. (I) Gefitinib promotes IL-1β release by stimulating the NLRP3 inflammasome. (II) Gefitinib promotes poly-ADP-ribosylation and release of HMGB1 via PARP-1 activation. Released-HMGB1 binds RAGE (receptor for advanced glycation endproducts) or TLR (Toll-like receptor) 2/4, which additively promotes IL-1β release [5].

Together our study has uncovered that gefitinib initiates sterile inflammation via two distinct mechanisms, and has also identified IL-1β and HMGB1 as critical determinants of gefitinib-induced inflammation (Figure 1) [5]. Interestingly, a recent study has identified papaverine as an inhibitor of HMGB1, and has demonstrated that papaverine can inhibit HMGB1-mediated inflammation [10]. Papaverine is commonly prescribed as an antispasmodic drug, which makes it possible to give an opportunity for drug repositioning. In addition, a series of NLRP3 inhibitors and IL-1β neutralizing antibodies are also potentially available as therapeutic agents for gefitinib-induced interstitial pneumonia. In any case, it is imperative to develop methods to prevent drug-induced interstitial pneumonia. To analyze the effects of pharmaceuticals on innate immune systems is expected to be a reliable approach to overcome drug-induced interstitial pneumonia.
